# Niacin skin flush and membrane polyunsaturated fatty acids in schizophrenia from the acute state to partial remission: a dynamic relationship

**DOI:** 10.1038/s41537-022-00252-w

**Published:** 2022-04-20

**Authors:** Ya-Hui Yu, Hui-Min Su, Sheng-Hsiang Lin, Po-Chang Hsiao, Yi-Ting Lin, Chih-Min Liu, Tzung-Jeng Hwang, Ming H. Hsieh, Chen-Chung Liu, Yi-Ling Chien, Chian-Jue Kuo, Hai-Gwo Hwu, Wei J. Chen

**Affiliations:** 1 Institute of Epidemiology and Preventive Medicine, College of Public Health, National Taiwan University, Taipei, Taiwan; 2 Graduate Institute of Physiology, College of Medicine, National Taiwan University, Taipei, Taiwan; 3 Institute of Clinical Medicine and Department of Public Health, College of Medicine, National Cheng Kung University, Tainan, Taiwan; 4 Biostatistics Consulting Center, National Cheng Kung University Hospital, College of Medicine, National Cheng Kung University, Tainan, Taiwan; 5 Department of Psychiatry, College of Medicine and National Taiwan University Hospital, National Taiwan University, Taipei, Taiwan; 6 Department of General Psychiatry, Taipei City Psychiatric Center, Taipei City Hospital, Taipei, Taiwan; 7 Centers of Genomic and Precision Medicine, National Taiwan University, Taipei, Taiwan; 8 Centers for Neuropsychiatric Research, National Health Research Institutes, Zhunan Town, Miaoli County Taiwan

**Keywords:** Molecular neuroscience, Schizophrenia, Biomarkers

## Abstract

Despite the consistent finding of an attenuated niacin-induced flush response in schizophrenia, its long-term stability and relationship to the membrane polyunsaturated fatty acid (PUFA) levels remain unknown. We conducted niacin skin tests and measured the membrane PUFAs using gas chromatography among 46 schizophrenia inpatients and 37 healthy controls at the baseline and the 2-month follow-up. Attenuated flush responses were persistently observed in schizophrenia patients in both acute and partial remission states, whereas an increased flush response was found in the controls. A persistent decrease in both dihomo-gamma-linolenic acid and docosahexaenoic acid and an increased turnover of arachidonic acid (ARA) via endogenous biosynthesis were found in schizophrenia patients. A composite niacin flush score by combining those with a control-to-case ratio of >1.4 (i.e., scores at 5 min of 0.1 M, 0.01 M, and 0.001 M + 10 min of 0.01 M and 0.001 M + 15 min of 0.001 M) at the baseline was correlated positively with ARA levels among controls but not among schizophrenia patients, whereas the flush score at the 2-month follow-up was correlated positively with ARA levels among patients. The 2-month persistence of attenuated niacin-induced flush response in schizophrenia patients implies that the niacin skin test might tap a long-term vulnerability to schizophrenia beyond acute exacerbation.

## Introduction

An attenuated niacin-induced flush response in schizophrenia has exhibited several characteristics that render it a potential endophenotype of schizophrenia^1–3^. The prevalence of the niacin response abnormality was higher in schizophrenia patients^4–6^, first-episode psychosis patients^7^, and ultra-high risk patients^8^ than in healthy controls. An attenuated niacin flush response has also been found in nonpsychotic first-degree relatives of schizophrenia patients^9–11^, and greater familial loading of schizophrenia was associated with more impairment in the flush response^12^. A genome-wide linkage scan of the niacin skin flush response among families of siblings co-affected with schizophrenia has led to some genetic linkage signals^13^. Nevertheless, whether the impairment in the niacin flush response is state-independent, another important characteristic for an endophenotype^14^, has received relatively less attention^3^. Repeated oral administration of nicotinic acid in healthy individuals exhibited tolerance within 2 days^15^. Only a few studies of small sample sizes examined whether the niacin response abnormality subsided after therapeutic intervention^16–18^. Thus, a prospective follow-up of niacin skin flush in patients with schizophrenia is warranted to clarify the stability of the niacin response abnormality.

The niacin-induced flush is exerted via a prostaglandin D2-related microvasodilation pathway^19,20^, triggered by its binding to a G-protein-coupled receptor on epidermal Langerhans cells^21^ to stimulate phospholipase A2 (PLA2) to release arachidonic acid (ARA) from cell membrane phospholipids^22^, which is further metabolized into prostaglandin molecules by cyclooxygenase-2 (COX-2)^23^. As postulated in the membrane hypothesis of schizophrenia, the loss of polyunsaturated fatty acids (PUFA), including ARA, results in membrane rigidity and can alter the conformation and functioning of proteins, receptors, and ion channels^24,25^. Dysfunctional ARA signaling, such as abnormal activities of PLA2 or COX-2 (refs. ^17,26^) and fatty acids composition of red blood cell (RBC) membranes^27,28^ have been implicated in schizophrenia patients. However, the relations of the genetic variants encoding the PLA2/COX-2 enzymes to the abnormal niacin response in schizophrenia is complex. When the distributions of the genetic variants of *PLA2G6* (encoding calcium-independent PLA2 beta) were compared between schizophrenia patients and controls, one study in Brazilians^29^ found a significant difference but three studies in Chinese^30^ or Croatians^31,32^ did not. When similar comparisons were conducted for *PLA2G4C* (encoding cytosolic PLA2 gamma), two studies in Chinese found significant differences^30,33^ but one study in Croatians did not^32^, although the latter did find a significant difference in *PLA2G6* and *PLA2G4C* genotype combination distributions^32^. Meanwhile, the niacin response in schizophrenia patients was associated not with the genetic variants of *PLA2G6* and *PLA2G4C*^34^ but with the variants of *PLA2G4A* (encoding cytosolic PLA2) and *PTGS2* (encoding COX-2)^35^. Two meta-analyses found the levels of docosapentaenoic (n-3) acid (DPA), docosahexaenoic (n-3) acid (DHA), and ARA of RBCs were decreased in medication-naïve patients, and a smaller reduction was also shown in medicated patients, though substantial heterogeneity among studies was noted^36,37^. Genetic studies found that desaturases, including delta-5 (D5D) and delta-6 (D6D) desaturase, play essential roles in the endogenous synthesis of ARA, whereas dietary intakes were the major source for DHA^38^.

The niacin response abnormality may reflect dysfunctional ARA-related signaling in schizophrenia patients. When schizophrenia patients took oral niacin, patients with the absence of a flush response showed reduced RBC levels of ARA and DHA^16^. Three studies applying topical niacin patches to schizophrenia patients failed to find any correlation between a niacin response abnormality and RBC membrane ARA levels^35,39,40^, though one of them did find such correlation in healthy controls^40^. Hence, the niacin response abnormality might be associated with a homeostatic imbalance in schizophrenia patients. Seldom examined is whether an acute state of schizophrenia could affect the relationship between the niacin-induced flush response and RBC membrane ARA levels.

To fill in these gaps in the literature, we conducted a follow-up study of schizophrenia patients at acute admission and partial remission 2 months later, as well as obtained similar measurements 2 months apart in a group of sex- and age-matched healthy controls. Specific aims of the study were to investigate the following: (1) whether the niacin flush response in the acute phase of schizophrenia at admission was attenuated compared to the response in healthy controls, and whether the attenuation would diminish at partial remission; (2) whether schizophrenia patients had altered RBC membrane levels of PUFAs or frequencies of the genetic variants of *PLA2G6* for calcium-independent PLA2 or *PLA2G4A* for cytosolic PLA2, compared to healthy controls, and whether the altered levels persisted at partial remission; and (3) whether there were correlations between the niacin-induced flush response and RBC membrane levels of PUFAs among schizophrenia patients in the acute phase at admission and in partial remission, and, for comparison, among healthy controls.

## Results

### Sample characteristics

Among 48 people with schizophrenia and 37 age- and sex-matched healthy volunteers recruited for this study, two patients were excluded from final analysis because their fatty acid analysis was not available. For the two SNPs genotyped in this study, *PLA2G6* rs4375 passed the quality control whereas *PLA2G4A* rs10798059 did not and hence only the former is reported here. Both the schizophrenia patients (*n* = 46) and healthy controls (*n* = 37) were similar in the distribution of sex, age, length of follow-up, and the genotypes of *PLA2G6* rs4375, but the patients had a higher prevalence of tobacco smoking than the controls (Table 1). Among the schizophrenia patients recruited, only four were at first onset with minimal treatment with antipsychotics while the others had varying histories of treatment for the illness. Since the two groups were frequency-matched with age and sex, we adjusted for potential confounders in comparing patients with controls using a propensity score consisting of age, sex, and smoking. From the baseline at admission to the follow-up at 8 weeks later or on the first out-patient follow-up if the patient had been discharged by 8 weeks, the Positive and Negative Syndrome Scale (PANSS) total score as well as its subscales scores of schizophrenia patients showed significant reduction. Hence, for simplicity the two time points were referred to as the baseline (acute) and approximately 2-month follow-up (partial remission) of schizophrenia patients, respectively, in the subsequent text.

**Table 1:** Sociodemographic and clinical characteristics of the participants in this study.

	Control (*n* = 37)	Schizophrenia (*n* = 46)
	*N* (%)	*N* (%)
Male	14 (37.8)	24 (52.2)
Smoking	6 (16.7)	22 (47.8)^a^
Allergy	6 (16.2)	4 (8.7)
Coffee	15 (40.5)	11 (23.9)
Drug		
First generation	–	6 (14.0)
Second generation	–	22 (51.2)
Mixed	–	15 (34.8)
*PLA2G6* genotype (rs4375)^b^		
TT	19 (52.8)	23 (51.1)
CT	15 (41.7)	19 (42.2)
CC	2 (5.5)	3 (6.7)
	Mean (SD)	Mean (SD)
Age	39.8 (11.8)	40.5 (10.8)
Follow-up interval (day)	72.4 (17.1)	68.8 (21.4)
Age of onset		23.8 (7.0)
Duration of illness		17.2 (10.5)
PANSS at T1 (baseline)	–	
Total	–	76.4 (21.2)
Positive	–	19.8 (4.7)
Negative	–	17.2 (8.2)
General psychopathology	–	33.9 (10.6)
PANSS changes at T2^c^	–	
Total	–	−12.7 (16.0)^d^
Positive	–	−4.9 (5.6)^d^
Negative	–	−1.8 (6.2)
General psychopathology	–	−6.0 (7.5)^d^

^a^*p* < 0.05 by *χ*^2^ test (compared with the control group).

^b^Information on the genotype of one control and one patient was missing.

^c^Eight weeks after the baseline or on the first outpatient follow-up if the patient had been discharged by 8 weeks, with 26 patients having the second PANSS rating.

^d^*p* < 0.05 by paired *t*-test (compared with the scores in the acute phase).

### Longitudinal pattern of niacin-induced flush

**Fig. 1:**
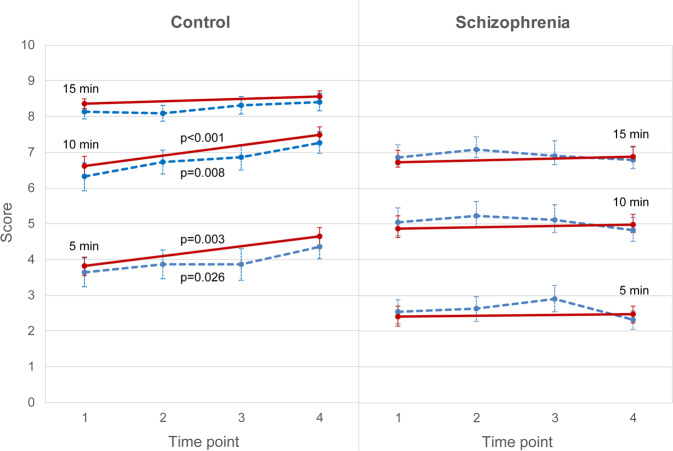
Longitudinal pattern of niacin-induced flush. Mean niacin flush score that was summed over the three concentrations for ratings at 5, 10, and 15 min, respectively, at different time points in **a** healthy controls and **b** schizophrenia patients, with error bars representing standard error. Time 1 = baseline, time 2 = week 2, time 3 = week 4, and time 4 = week 8 or on the first outpatient follow-up if the patient had been discharged by 8 weeks. Solid lines represent the scores of all participants who had flush scores at both time 1 and time 4 (*n* = 37 for controls and 46 for schizophrenia) and dashed lines the scores of a subgroup of participants who had flush scores at all four time points (*n* = 22 for controls and 35 for schizophrenia). For those summed niacin flush scores that changed significantly with time in a mixed-effects model were denoted with *p* values.

Schizophrenia patients had lower mean niacin flush scores at a variety of time–concentration combinations than healthy controls at both baseline and the 2-month follow-up (Supplementary Table S1). In comparing the niacin flush score at the two time points, we summed up the rating over the three concentrations for different time points. As shown in Fig. 1, the healthy controls showed a significant increase in flush scores from baseline to the 2-month follow-up for the summed ratings at 5 and 10 min, but not at 15 min, after correction for multiple testing. In contrast, schizophrenia patients did not show any increase in flush score from admission to discharge 2 months later. Furthermore, the patients’ flush scores were significantly lower than the controls’ scores for all summed scores across concentrations at 5, 10, and 15 min.

For participants who had additional niacin ratings at weeks 2 and 4, displayed as the dotted line in Fig. 1, the niacin ratings on four occasions (week 0, week 2, week 4, and week 8) of controls (*n* = 22) exhibit an increasing trend for ratings at 5 and 10 min, whereas those of schizophrenia patients (*n* = 35) did not. The results of the mixed-effects regression analysis confirmed that there was a significant group effect plus a significant interaction between group and time for ratings at 5 and 10 min, respectively.

To derive a composite niacin score that maximized the contrast between schizophrenia patients and healthy controls at baseline, we calculated the ratio of niacin score at each time–concentration point (Supplementary Table S2). We combined the ratings of those time–concentration points that have a ratio of >1.40, i.e., scores at (5 min of 0.1 M, 0.01 M, and 0.001 M) + (10 min of 0.01 M and 0.001 M) + (15 min of 0.001 M), to construct the composite niacin flush score.

We then examined whether the composite niacin flush score at baseline was associated with *PLA2G6* genotype (rs4375), which was grouped into the presence of C (i.e., CC or CT) and absence of C (i.e., TT), separately for schizophrenia patients and healthy controls. Comparing the mean of two genotype groups with adjustment using a propensity score consisting of age, sex, and smoking, neither schizophrenia patients (6.55 [SD = 1.02] with *n* = 22 vs. 6.13 [SD = 0.95] with *n* = 23, *p* = 0.79) nor healthy controls (9.94 [SD = 0.76] with *n* = 17 vs. 10.21 [SD = 0.82] with *n* = 19, *p* = 0.27) reached statistical significance.

### Membrane PUFAs and schizophrenia

The levels of 24 fatty acids in the RBC membrane are listed in Supplementary Table S3 for controls and Supplementary Table S4 for schizophrenia patients. Since this study was focused on PUFAs, only this part of the profile is reported in Table 2. We calculated the average of controls’ measurements at baseline and the 2-month follow-up as the pooled reference. Compared to the pooled reference, schizophrenia patients at admission had increased levels of gamma-linoleic acid (GLA; effective size = 0.6) and adrenic acid (effective size = 0.8), but had decreased levels of dihomo-gamma-linolenic acid (DGLA; effective size = −0.7) and docosahexaenoic acid (DHA; effective size = −0.6) after correction for multiple testing (Table 2). At the 2-month follow-up, schizophrenia patients had decreased levels of DGLA (effective size = −0.6), DHA (effective size = −0.8), and sum of n-3 fatty acids (effective size = −0.7) after correction for multiple testing.

**Table 2:** Fatty acid composition (%) of red blood cells at the acute (T1) and partial remission stage (T2) of the schizophrenia patients as compared to the pooled controls.

	Schizophrenia (*n* = 46)						Pooled controls^c^	
	T1 at the baseline			T2 at the follow-up^b^			(*n* = 37)	
Fatty acids	Mean	(SD)	ES^a^	Mean	(SD)	ES	Mean	(SD)
n-6 PUFAs								
C18:2 (linoleic acid)	15.99	(2.38)	−0.2	15.77	(2.52)	−0.3	16.33	(1.64)
C18:3 (γ-linolenic acid)	0.42^**d**^	**(0.24)**	0.6*	0.35	(0.11)	0.4	0.31	(0.06)
C20:2 (eicosadienoic acid)	**0.44**^**d**^	**(0.11)**	**0.4**	0.43	(0.10)	0.4	0.40	(0.06)
C20:3 (dihomo gamma linolenic acid)	**0.93**^**d**^	**(0.33)**	−0.7*	**0.98**^**d**^	**(0.31)**	−0.6*	1.13	(0.18)
C20:4 (arachidonic acid)	12.22	(2.22)	−0.2	12.16	(2.61)	−0.2	12.57	(1.22)
C22:2 (docosadienoic acid)	**0.15**^**d**^	**(0.06)**	**0.4**	**0.17**^**d**^	**(0.17)**	**0.3**	0.13	(0.05)
C22:4 (adrenic acid)	**2.87**^**d**^	**(0.94)**	0.8*	2.35	(0.72)	0.3	2.13	(0.57)
C22:5 (docosapentaenoic acid)	0.51	(0.15)	−0.2	0.52	(0.23)	−0.1	0.54	(0.15)
Sum of n-6 fatty acids	33.52	(3.20)	0.0	32.73	(4.14)	−0.2	33.53	(2.10)
n-3 PUFAs								
C18:3 (α-Linolenic acid)	0.29	(0.11)	0.1	0.28	(0.08)	0.0	0.28	(0.09)
C20:5 (eicosapentaenoic acid)	0.57	(0.30)	−0.2	0.52	(0.22)	−0.4	0.62	(0.26)
C22:5 (docosapentaenoic acid)	1.75	(0.42)	0.1	1.66	(0.43)	−0.2	1.73	(0.41)
C22:6 (docosahexaenoic acid)	**4.37**^**d**^	**(1.23)**	−0.6*	**4.11**^**d**^	**(1.23)**	−0.8*	4.98	(0.80)
Sum of n-3 fatty acids	**6.98**^**d**^	**(1.75)**	**−0.4**	**6.57**^**d**^	**(1.64)**	−0.7*	7.61	(1.11)
Sum of polyunsaturated fatty acids	40.50	(4.24)	−0.2	39.30	(5.11)	−0.5	41.14	(1.85)

^a^ES: effect size calculated as [(mean difference between schizophrenia and pooled controls)/SD (the whole sample)].

^b^Eight weeks after the baseline or on the first outpatient follow-up if the patient had been discharged by eight weeks.

^c^Pooled controls: mean fatty acids levels of controls at baseline and 2-month follow up.

^d^*p* < 0.05 for comparison with the pooled controls in linear regressions adjusting for propensity score consisting of age, sex, and smoking.

*Significant after correction for multiple testing by false discovery rate.

Since the level of ARA (20:4n-6) might be influenced by endogenous biosynthesis, i.e., being converted to adrenic acid (22:4n-6) via elongation and being replenished from DGLA (C20:3n-6) via desaturation, we then examined the relationship of the levels of ARA to that of its product (adrenic acid) as well as its precursor (DGLA) (Supplementary Fig. S1). For controls at baseline, levels of ARA were correlated neither with adrenic acid nor with DGLA. For schizophrenia patients, ARA was correlated positively with adrenic acid both in the acute state (*r* = 0.487) and at partial remission (*r* = 0.574), and was correlated negatively with DGLA at partial remission (*r* = −0.606) but not in the acute state. When Spearman correlation was used for these analyses, the results remain the same (Supplementary Table S5).

To further explore the mechanisms underpinning the altered levels of fatty acids among schizophrenia patients, we estimated desaturase activities by means of product/precursor ratios of PUFA, following a previous study^41^. The results revealed that schizophrenia patients at both baseline and the 2-month follow-up had higher D5D and D6D activities than controls (Table 3).

**Table 3:** Desaturase activities estimated from RBC in schizophrenia patients and controls.

	Schizophrenia (*N* = 46)		Pooled controls (*N* = 37)
	Baseline	2-month follow-up	
Desaturase activities^a^	Mean (SD)	Mean (SD)	Mean (SD)
Delta 6 desaturase (×10^3^)	27.6 (20.6)*	23.9 (11.7)*	19.2 (5.4)
Delta 5 desaturase	14.5 (5.4)*	13.8 (4. 9)*	11.4 (2.0)

^a^Delta-5 desaturase activity was estimated from the ratio of (20:4 n-6)/(20:3 n-6), while delta-6 desaturase activity was from the ratio of (18:3 n-6)/(18:2 n-6).

^*^ *p* < 0.05 for comparison with the pooled controls in linear regressions adjusting for propensity score consisting of age, sex, and smoking.

### PUFAs and the niacin flush response

Then, we examined the relations of ARA, DGLA, and adrenic acid to the composite niacin flush score (Supplementary Table S6). Both ARA and DGLA exhibited correlations with the niacin composite score of controls at baseline, and DGLA exhibited correlations with the niacin composite score of schizophrenia patients at partial remission; meanwhile, adrenic acid did not have any correlation with the composite niacin flush score.

**Fig. 2:**
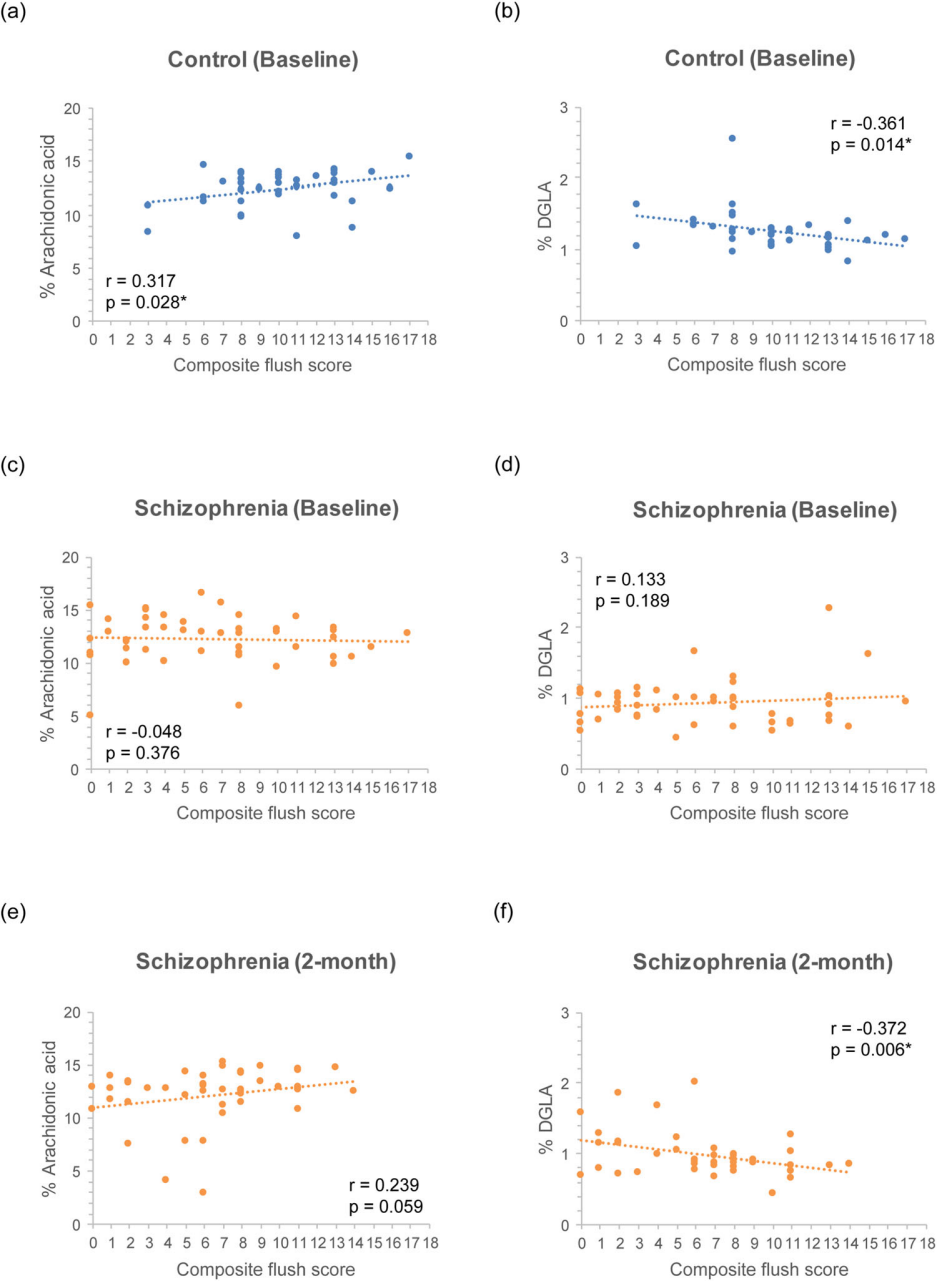
PUFAs and the niacin flush response. Pearson correlations between the composite flush score of the niacin skin test and the level of arachidonic acid and dihomo-gamma-linolenic acid (DGLA) for healthy controls (*n* = 37) at baseline (**a**, **b**) as well as schizophrenia patients (*n* = 46) at baseline (**c**, **d**) and at a 2-month follow-up (**e**, **f**).

Figure 2 plots the niacin composite score with ARA and DGLA, respectively. For controls at baseline, ARA levels were positively correlated with the composite niacin score (*r* = 0.317, *p* = 0.028), while DGLA levels were negatively correlated with the composite niacin score (*r* = −0.361, *p* = 0.014). In contrast, for schizophrenia patients at baseline, neither ARA nor DGLA exhibited correlations with the composite niacin flush score. However, for schizophrenia patients at the 2-month follow-up, the composite niacin flush score had a borderline positive correlation with ARA (*r* = 0.239, *p* = 0.059) and a negative correlation with DGLA (*r* = −0.372, *p* = 0.006). When Spearman correlation was used for these analyses, the results remain the same (Supplementary Table S7).

## Discussion

In this prospective study, healthy controls exhibited enhanced niacin-induced flush at the 2-month follow-up, whereas schizophrenia patients did not have significant changes in their flush response from the baseline to the 2-month partial remission, remaining attenuated at both time points. Both groups did not differ in the genotype distribution of *PLA2G6* rs4375, and participants’ niacin responses were not associated with this gene’s variants within each group. Lower RBC membrane levels of DGLA and DHA in patients as compared to the controls were consistently observed at both time points. In addition, increased levels of the precursor (DGLA) and product (adrenic acid) of the endogenous biosynthesis of ARA were observed in schizophrenia patients only at admission. A composite score of the niacin flush response was positively correlated with ARA and negatively correlated with DGLA levels in the controls at baseline, but failed to show such correlations in schizophrenia patients. Nevertheless, at the partial remission, the niacin flush response in schizophrenia patients became weakly correlated with ARA and negatively correlated with DGLA levels. These findings provide support for elevated turnover of ARA signaling as the pathophysiology underpinning the attenuated flush response to niacin in schizophrenia and its dynamic relationship to membrane PUFAs.

A surprising finding is that healthy controls had increased niacin-induced flush response to the repeated niacin skin tests at the 2-month follow-up. This is different from the finding of a previous study of healthy volunteers, in which the flush response to oral niacin dosing diminished within 2 days^15^. In contrast, schizophrenia patients’ flush response to the repeated niacin skin tests remained attenuated and unchanged during the 2-month period, even when patients already had partial remission in symptoms. Previous stability studies in schizophrenia patients used a binary rating of the flush after oral niacin intake^18^ or application of a topical niacin patch^17^. Despite the differences in the method of administration and scoring, approximately 70–80% of the nonflushers among schizophrenia patients persisted to be so 2 (ref. ^17^) or 3 months^18^ later. Since the dermal niacin receptor that binds niacin is located in Langerhans cells^21^, there may be immune memory by these immune cells or other intermediates in the cascade after the initial activation by the niacin skin patch in healthy controls. Meanwhile, schizophrenia patients might have certain immunological aberrations that are related to the persistence of attenuated niacin-induced flush response. Increasing evidence indicates that there are immune system disturbances in schizophrenia^42,43^, e.g., autoantibodies to neurotransmitter receptors^44–46^ or inflammation^47,48^. Whether these immune system disturbances are associated with the aberrations in niacin-induced flush response in schizophrenia warrants future investigation.

One potential contributor to the niacin-induced flush abnormality in schizophrenia examined in this study is the genetic variants of *PLA2G6* (rs4375). We found that its genotype distributions were not different between schizophrenia patients and healthy controls, which is different from the association found in Brazilians^29^ but consistent with studies in Chinese^30^ or Croatians^31,32^. Furthermore, we also found the niacin-induced flush response was not associated with genotypes of *PLA2G6*, regardless of schizophrenia patients or healthy controls. This is consistent with a previous study in Croatian patients with schizophrenia^34^. Nevertheless, there are other PLA2 genes that were found to be associated with the risk of schizophrenia, e.g., *PLA2G4C*^29,30,33^, or the niacin-induced flush response in schizophrenia patients, e.g., *PLA2G4A* and *PTGS2* (ref. ^35^). In addition, when the plasma levels of different forms of PLA2 were measured, the levels of calcium-independent PLA2 were higher in both schizophrenia patients^49^ and ultra-high risk individuals^50^ than in controls, whereas the level of cytosolic PLA2 were higher only in individuals with ultra-high risk as compared to controls^50^. Whether the niacin-induced flush abnormality could be attributed to the genetic polymorphisms of other PLA2 genes or the altered levels of PLA2 warrants future investigation.

In comparing the fatty acid composition between groups or time points, we reported in the form of percentage of total fatty acids rather than the quantification of the actual amount (weight) of each fatty acid that is present in the RBC membrane. Regarding the RBC membrane’s PUFAs, schizophrenia patients had persistent decrease at both baseline and the 2-month partial remission in the levels of DHA, a n-3 PUFA with main source from dietary intake, and DGLA, a n-6 PUFA and precursor of the biosynthesis of ARA. The lowered level of DHA is consistent with the findings from two meta-analyses^36,37^. Unlike ARA, which is released from membrane preferentially by cytosolic PLA2 and has prooxidant and proinflammatory properties, DHA is predominantly released by calcium-independent PLA2 and has antioxidant and anti-proinflammatory properties^51,52^. In addition to lower dietary intake of PUFAs^53^, increased calcium-independent PLA2 levels might also contribute to the lowered level of RBC membrane’s DHA^49^.

Meanwhile, the persistent decrease in the level of DGLA in schizophrenia patients should be understood in the context of the endogenous biosynthesis of ARA^38^. Under this circumstance, the endogenous synthesis of ARA was not apparent in healthy controls, indicated by their lack of ARA’s correlation with its precursor, but was active in schizophrenia, with ARAs having a negative correlation with DGLA. One reason that schizophrenia patients had active endogenous synthesis of ARA is due to an increased turnover of membrane ARA as a result of increased cytosolic PLA2 activity. Because of this replenishment of ARA via endogenous synthesis, we did not find a decrease in the membrane levels of ARA in schizophrenia patients, which was also reported in some previous studies^40,54^. Another explanation is the possible normalization effect of long-term anti-psychotic treatment^55^. Since the majority of our patients were not in their first episode, we could not rule out this possibility.

Furthermore, the D5D desaturase activity, which turns DGLA into ARA, was found to be higher in schizophrenia patients than in controls at both time points in this study. Meanwhile, the membrane level of adrenic acid in schizophrenia patients was elevated in the acute state but not at the 2-month partial remission. Consequently, our findings indicate that the temporal availability of membrane ARA in schizophrenia patients might be more at the partial remission than in the acute state. A previous study of small sample size (20 schizophrenia patients and 20 controls) also reported a tendency for schizophrenia patients to have an increased membrane levels of adrenic acid^40^. Intriguingly, our results showed that patients’ DGLA levels had a negative correlation with that of ARA only at the 2-month partial remission, when a continuous replenishment of ARA from DGLA was in synchronization with a slowdown in the turnover of ARA into adrenic acid at this time point.

The positive correlation between the niacin-induced flush response with the membrane levels of ARA in heathy controls in this study was consistent with a previous study showing a positive correlation between Doppler-measured blood flow and levels of ARA^40^. However, the relations of the niacin-induced flush response to ARA’s precursor or product varied across studies. The niacin-induced flush response in this study was correlated with DGLA levels but not with adrenic acid levels in healthy controls at baseline, whereas the Doppler-measured blood flow in the previous study^40^ showed trends towards correlation with adrenic acid levels but not with DGLA levels in healthy controls. The discrepancies might be due to varying enzymatic activities of D5D in the endogenous biosynthesis of ARA for patients in different clinical stages.

Despite that schizophrenia patients had similar levels of ARA in both the acute and partial remission states, the correlation between ARA level and the composite niacin-induced flush score was lost in the acute state but regained weakly at the 2-month partial remission. We postulated that only when the temporal availability of ARA increased as a result of balance between replenishment of ARA from DGLA with the turnover of ARA into adrenic acid, the niacin-induced flush response had a positive correlation with ARA levels and a negative correlation with DGLA levels. Future studies are warranted to investigate whether the synchronization in the turnover of both DGLA and ARA on the RBC membranes is an indicator of partial remission.

Alongside the increased turnover of ARA, a n-6 PUFA, via endogenous biosynthesis, schizophrenia patients exhibited a lowered membrane level of DHA, a n-3 PUFA relying mainly on dietary intake, at both the baseline and the 2-month partial remission. This might reflect an increased turnover of DHA due to genetic predisposition^56^, lowered dietary intake^57^, or elevated oxidative stress^55,58,59^ in schizophrenia patients. Regardless of the causes, supplementation with DHA might have a beneficial effect on patients’ improvement in psychotic symptoms. Indeed, supplementation of antipsychotics with n-3 PUFAs has been shown to improve symptoms in schizophrenia patients^52,60–62^. A recent Mendelian randomization analysis revealed that schizophrenia patients might have difficulty converting short-chain PUFA into long-chain PUFAs^63^.

A related question is whether the attenuated niacin-induced flush response existed in individuals at increased risk for schizophrenia. Previous family studies indicated that individuals at familial risk for schizophrenia did have increased risk of attenuated niacin-induced flush response^9–12^. However, the results in individuals at ultra-high risk for psychosis remain conflicting^8,64^. Furthermore, several randomized trials of large sample sizes^65–68^ failed to replicate an earlier report that supplementation of n-3 PUFAs could lower the risk of conversion to psychosis among individuals at ultra-high risk for psychosis^69^. Hence, the relationship between membrane levels of PUFAs and the symptoms of attenuated psychosis syndrome warrants further investigation.

Our results have implications for the niacin-induced flush abnormality as a biomarker for schizophrenia and help shed light on its underlying pathophysiology. First, the 2-month persistence of attenuated flush response in schizophrenia patients implies that the niacin skin test might tap a long-term vulnerability to schizophrenia beyond acute exacerbation. When schizophrenia patients underwent membrane lipid compositional changes from acute phase at baseline to partial remission at the 2-month follow-up, e.g., an increased level of adrenic acid, resumption of a negative correlation in levels between DGLA and ARA, and thus resulting in a correlation of the composite flush score with ARA (positively) and DGLA (negatively) as healthy controls at baseline. Second, the 2-month attenuated niacin-induced flush response in schizophrenia patients is accompanied by the persistent lowering of RBC membrane’s DGLA and DHA. DGLA is a n-6 PUFA that can be converted from GLA by elongation and, in addition to be converted into ARA by D5D desaturase, can be metabolized by COX-1 into prostaglandin E1, free radicals, and reactive oxygen species^70^. Meanwhile, DHA is a n-3 essential fatty acid that is released from membrane by iPLA2 and can be metabolized by lipoxygenases to antioxidative molecules, such as docosanoids, resolvins, and neuroprotectins^52^. Thus, a long-lasting vulnerability underlying schizophrenia tapped by the niacin skin test might be dysfunction in antioxidative and anti-proinflammatory mechanisms in schizophrenia patients^47,71,72^. Third, if that is the case, it stands to reason that once the levels of DGLA and DHA return to normal, which might render the niacin-induced flush response un-attenuated, a patient with schizophrenia might be in a state free of relapse in a relatively long period. At that time, the levels of prostaglandin E1, resolvins, and protectins, might also be back to normal. Thus, it is of importance to follow-up the niacin flush response in schizophrenia patients for longer terms to assess whether an increase of flush response is parallel to an increase in RBC membrane’s levels of DGLA and DHA as well as antioxidative and anti-inflmmatory molecules. Finally, in contrast to the complexity and high-cost in the measurement of RBC membrane’s PUFAs, the niacin skin test appears to be a simple, cheap, and fast test that taps the disturbance in membrane’s lipid homeostasis in schizophrenia and can be easily applied in clinical settings.

There were limitations in this study. First, our use of a 4-point Likert scale in measuring niacin-induced flush has relatively low resolution compared to Doppler-based blood flow. Nevertheless, our method allowed for testing in multiple concentrations and calculating a composite flush score to help enhance the discrimination of schizophrenia patients from healthy controls. Second, we did not have detailed food intake history of the participants and could not exclude the influence of food intake on the RBC membrane levels of PUFAs. There have been reports that schizophrenia patients had lower dietary intakes of PUFAs than controls^53,57,73,74^, though some studies reported an opposite direction^75^. Third, despite our statistical adjustment for tobacco smoking in comparing the niacin-induced skin flushing between schizophrenia patients and healthy controls, there might still be residual confounding since the former had a much higher prevalence of smoking than the latter. Cigarette smoke may directly contribute to oxidative stress and decreases in antioxidants^76,77^, and result in nicotine-induced inhibition of neuronal PLA2 activity^78,79^. Finally, a mixture of schizophrenia patients with varying numbers of psychotic episodes make it difficult to assess the influence of illness duration and antipsychotic treatment. Finally, the lack of associations between the levels of certain PUFAs and flush scores might be due to the small sample size of this study. A future study of larger sample size is warranted to validate the clues observed in this study.

In conclusion, this 2-month follow-up study found that unlike healthy controls’ enhanced flush response to the repeated niacin skin tests, schizophrenia patients’ flush response remained attenuated and unchanged at the 2-month follow-up. The difference in the niacin response could not attributed to the distribution of *PLA2G6* variants. In a comprehensive measurement of RBC membrane’s PUFAs, there were aberrations in the levels of both n-3 and n-6 PUFAs in schizophrenia patients, particularly a persistent decrease in DHA and an increased turnover of ARA via endogeneous biosynthesis. Furthermore, the niacin-induced flush response in schizophrenia had a dynamic relationship with the aberrant ARA signaling from the acute state to the 2-month partial remission. These findings warrant future investigation on how the dysfunctional regulation of membrane phospholipids can be predicted or corrected in schizophrenia patients at an early stage.

## Methods

### Participants

Our participants were from the Lipid Biology of Schizophrenia and Schizotypal Traits (LIBISS) project conducted at the Department of Psychiatry, National Taiwan University Hospital, NTUH Yun-Lin Branch, and Taipei City Hospital Song-De Branch, as described previously^80^. Briefly, a total of 48 people with schizophrenia (aged 23–66) and 37 age- and sex-matched healthy volunteers made up of students and the general population were recruited between August 2009 and July 2012. Patients meeting the DSM-IV criteria of schizophrenia were recruited for interviewing using the Chinese translation^81^ of the Diagnostic Interview for Genetic Studies (DIGS)^82^. Healthy volunteers were screened using the DIGS to confirm the absence of a current or previous psychiatric history. Exclusion criteria for the study were severe neurological abnormality, prominent substance use problem, or mental retardation. The study was approved by the Research Ethics Committee of National Taiwan University Hospital (200812089R). Each participant provided written informed consent after being given a complete description of the study.

### Measurements

For schizophrenia patients, the severity of their symptoms was assessed by their attending psychiatrist using the Positive and Negative Syndrome Scale (PANSS)^83^ at the baseline as well as 8 weeks later or on the first outpatient follow-up if the patient had been discharged by 8 weeks (referred to as the 2-month follow-up for simplicity). For both schizophrenia patients and healthy controls, we performed fasting blood sample collection and a niacin skin test at the baseline as well as the 2-month follow-up.

The PANSS consists of 30 items, each with a 7-point rating scale, and is classified into the positive symptom subscale (7 items), negative symptom subscale (7 items), and general psychopathology subscale (16 items), and its translation into a Mandarin Chinese version was shown to have good interrater reliability^84^, with intraclass correlation coefficients ranging from 0.64 to 0.96) in a later study^85^. All the participating psychiatrists had received relevant training on the use of the PANSS.

### Niacin skin test and visual rating scale

The niacin skin test was modified from Ward et al.^86^. We used laboratory filters saturated with aqueous methyl nicotinate (AMN) as patches to apply on each subject’s forearm using four patches: three contained 50 μl of different concentrations (0.001, 0.01, and 0.1 M) of AMN and the other one was a blank negative control. After 5 min exposure to AMN, the patches were removed. The flush reaction was rated at 5, 10, and 15 min following the application with a four-point scale (0 = no erythema, 1 = incomplete erythema, 2 = complete erythema, and 3 = erythema plus edema). For quality control, a photograph was taken at each rating for later cross-evaluation. To evaluate the trend in the niacin-induced flush over time, some of the participants (35 schizophrenia patients and 22 controls) underwent additional niacin skin tests at weeks 2 and 4, along with the baseline and week 8 (or on the first outpatient follow-up if the patient had been discharged by 8 weeks) tests.

The interrater reliability of the four-point scale in rating the niacin-induced skin flush was shown to be excellent between two psychiatrists in a preliminary study of 50 subjects (34 schizophrenic patients, 4 bipolar affective patients, and 12 normal controls) with the intraclass correlation coefficient ranging from 0.85 to 0.94 for different concentrations of niacin^87^. In a later study of 50 subjects (25 schizophrenic patients and 25 controls), five research assistants trained by the two psychiatrists performed the rating independently and their interrater reliability was shown to be good, with the intraclass correlation coefficient ranging from 0.69 to 0.76 (ref. ^11^). The research assistants of this study received similar training before they conducted the test independently.

### Fatty acid analysis

We used the method described by Moser et al.^88^ to prepare total lipid fatty acid methyl esters of plasma and RBCs. More details are provided in Supplementary Methods. Plasma layer and RBCs were first separated and stored at −80 °C until analysis. For the RBCs, after an overnight extraction, the lower layer was transferred to a clean tube. We then dried the sample under nitrogen. Then, 1 ml of methanol:dichloromethane (3/1, v/v) were added into 200 μl of the RBCs preparation. Mixed with 0.2 ml acetyl chloride, we heated the tube in a 75 °C oven for 1 h. We then used Agilent 7820 (Agilent Technology, Santa Clara, CA) for gas chromatography with flame ionization detector. The fatty acids peaks were determined by means of comparison of the retention times with those of a standard mixture of 37 FAME, PUFA2, and PUFA3 (Supelco/Sigma-Aldrich, Bellefonte, PA). The fatty acid composition was expressed as the percentage of the total fatty acid (% total fatty acids). The fatty acid analysis was not available for two patients, and hence the final sample size for this study was 46 patients and 37 controls.

### Genotyping

Genomic DNA samples extracted from venous blood using the QIAamp DNA Blood Mini Kit (Qiagen, Hilden, Germany) were subjected to genotyping using an ABI TaqMan 7900 system (Applied Biosystems, Foster City, CA) for two polymorphisms: (1) the C/T (*Avr*II polymorphic site; rs4375) in the fourth intron of *PLA2G6* and (2) the A/G (*Ban*I polymorphic site; rs10798059) in the first intron of *PLA2G4A*^29^. The results of rs10798059 failed to distinguish genotypes clearly and were not included for analysis.

### Data analysis

The sample size of this study for detecting group differences in niacin-induced flush response between schizophrenia patients and healthy controls was estimated under the following assumptions: power = 0.8, *α* = 0.05, equal number in each group, the proportion of subjects with absent flush response being 10% for healthy controls and 25% for schizophrenia patients, and each participant receiving repeated measurements of four times with the correlation structure being first-order autoregressive of *ρ*. Then the sample size was 72 if *ρ* = 0.2 and 94 if *ρ* = 0.4.

Group comparisons were performed using the *χ*^2^ test for categorical variables and t-test for continuous variables, if indicated. The mixed-effects models using the procedure of PROC MIXED, SAS Version 9.4 (SAS Institute, Cary, NC) were applied to examine the influence of time, dose, and group on the flush scores, adjusting for other covariates using the propensity score method.

Multivariable linear regression analyses were used to compare fatty acid levels between two groups with adjustment using the propensity score. We corrected for multiple testing using false discovery rate. Effect size was calculated as the mean difference between two groups divided by the SD of the whole sample.

Within each group of controls and patients, Pearson correlation coefficients were used to examine the correlation between flush scores and fatty acid composition. Since the primary hypothesis was directional^40^, i.e., higher AA or adrenic acid levels would correlate with more niacin response whereas higher DGLA levels would correlate with less niacin response, the significance testing of the correlation was one-tailed. For sensitivity analysis, we used Spearman correlation to repeat this part of the analysis. A *p* value less than 0.05 was considered significant.

## Supplementary information

Supplementary data

## Data Availability

The datasets used and analyzed in the current study are not publicly available due to conditions on participant consent and other ethical restrictions. However, the data that support the findings of this study are available from the corresponding author upon reasonable request.

## Code availability

The SAS code that supports the findings of this study are available from the corresponding author upon reasonable request.
