# Supplementary material for: Niacin skin flush and membrane polyunsaturated fatty acids in schizophrenia from the acute state to partial remission: a dynamic relationship
Source: Schizophrenia (Heidelb). 2022 Apr 20;8(1):38. doi: 10.1038/s41537-022-00252-w (PMC9261101; doi:10.1038/s41537-022-00252-w)
Supplement: Supplementary file 1 — Supplementary data [file 41537_2022_252_MOESM1_ESM.doc]

**Supplementary data**

**Supplementary methods.**

**Supplementary figures and tables**

**Fig. S1.** Pearson correlations between the level of arachidonic acid and that of its precursor (dihomo-gamma-linolenic acid, DGLA) and product (adrenic acid) for healthy controls (n = 37) at baseline (a, b) as well as schizophrenia patients (n = 46) at baseline (c, d) and at a 2-month follow-up (e and f).

**Table S1**. Niacin scores at a series of time-concentration points in controls and schizophrenia patients at two time points.

**Table S2**. Niacin scores at a series of time-concentration points at baseline in controls and schizophrenia patients.

**Table S3**. Fatty acid composition (%) of red blood cell at baseline and 2-month follow-up of control group.

**Table S4**. Fatty acid composition (%) of red blood cell at baseline and 2-month follow-up of schizophrenia patients versus the pooled controls.

**Table S5**. Pearson correlations and Spearman correlations between levels of arachidonic acid (ARA) and its product (adrenic acid) and precursor (dihomo gamma linolenic acid, DGLA), respectively, for controls at the baseline, schizophrenia patients at the baseline, and schizophrenia patients at the 2-month follow up.

**Table S6**. Pearson correlations between n-6 fatty acids (arachidonic acid, its precursor dihomo gamma linolenic acid, and product adrenic acid) and the composite niacin scores for controls at the baseline, schizophrenia patients at the baseline, and schizophrenia patients at the 2-month follow up.

**Table S7**. Spearman correlations between n-6 fatty acids (arachidonic acid, its precursor dihomo gamma linolenic acid, and product adrenic acid) and the composite niacin scores for controls at the baseline, schizophrenia patients at the baseline, and schizophrenia patients at the 2-month follow up

**Supplementary methods**

*Fatty acid analysis*

Approximately 7 mL whole blood was collected into an EDTA-containing tube for each subject who had overnight fasting. The collected blood was kept on ice until centrifuged at 2000rpm for 30 min at 4°C. Then plasma layer and RBCs were separated and stored at -80°C until analysis.

We used the method described by Moser et al. (1999) to prepare total lipid fatty acid methyl esters of RBCs. We used 300 microliter RBCs and then added 3mL chloroform and 2mL 0.4%NaCl with 20 min sonication. After an overnight extraction at 4°C, we centrifuged the sample at 3000 rpm for 10 min. Taking the lower layer and transferring it to a clean tube, we then dried the sample under nitrogen. Then, 1 mL of methanol:dichloromethane (3/1, v/v) were added into 200 μL of the RBCs preparation. Mixed with 0.2 mL acetyl chloride, we heated the tube in a 75°C oven for 1 hour.

After the tube cooled down, 4 mL of 7% potassium carbonate and 2mL of hexane were added to extract methyl esters of fatty acids. We centrifuged the tube at 3000 rpm for 10 min and dried the upper layer of the sample under nitrogen. According to the concentration of samples, approximately100 μL of hexane was added and transferred to the vial for gas chromatography (7820 System, Agilent Technology, Santa Clara, CA) analysis.

Agilent 7820 gas chromatography used ﬂame-ionization detector on a polar column (100 m x 0.25 mm x 0.20 um, Supelco 2560 column) with nitrogen (99.99%) as carrier gas. The oven temperature program was set at 60°C for 2 min, then increased by 25°C per min to 160°C, then by 2°C per min to 240°C, and held at 240°C for 20 min. Finally, the oven temperature increased by 5°C per min to 245°C and held for 10 min. The total time of the program was 77 min. The fatty acids peaks were determined by means of comparison of the retention times with those of a standard mixture of 37 FAME, PUFA2, and PUFA3 (Supelco/Sigma-Aldrich, Bellefonte, PA). The fatty acid composition was expressed as the percentage of the total fatty acid (% total fatty acids). The fatty acid analysis was not available for two patients, and the final sample size for this study was 46 patients and 37 controls.

**Reference**

Moser A B, Jones D S, Raymond G V& Moser H W. Plasma and red blood cell fatty acids in peroxisomal disorders. *Neurochem Res* **24**. 187-197 (1999)


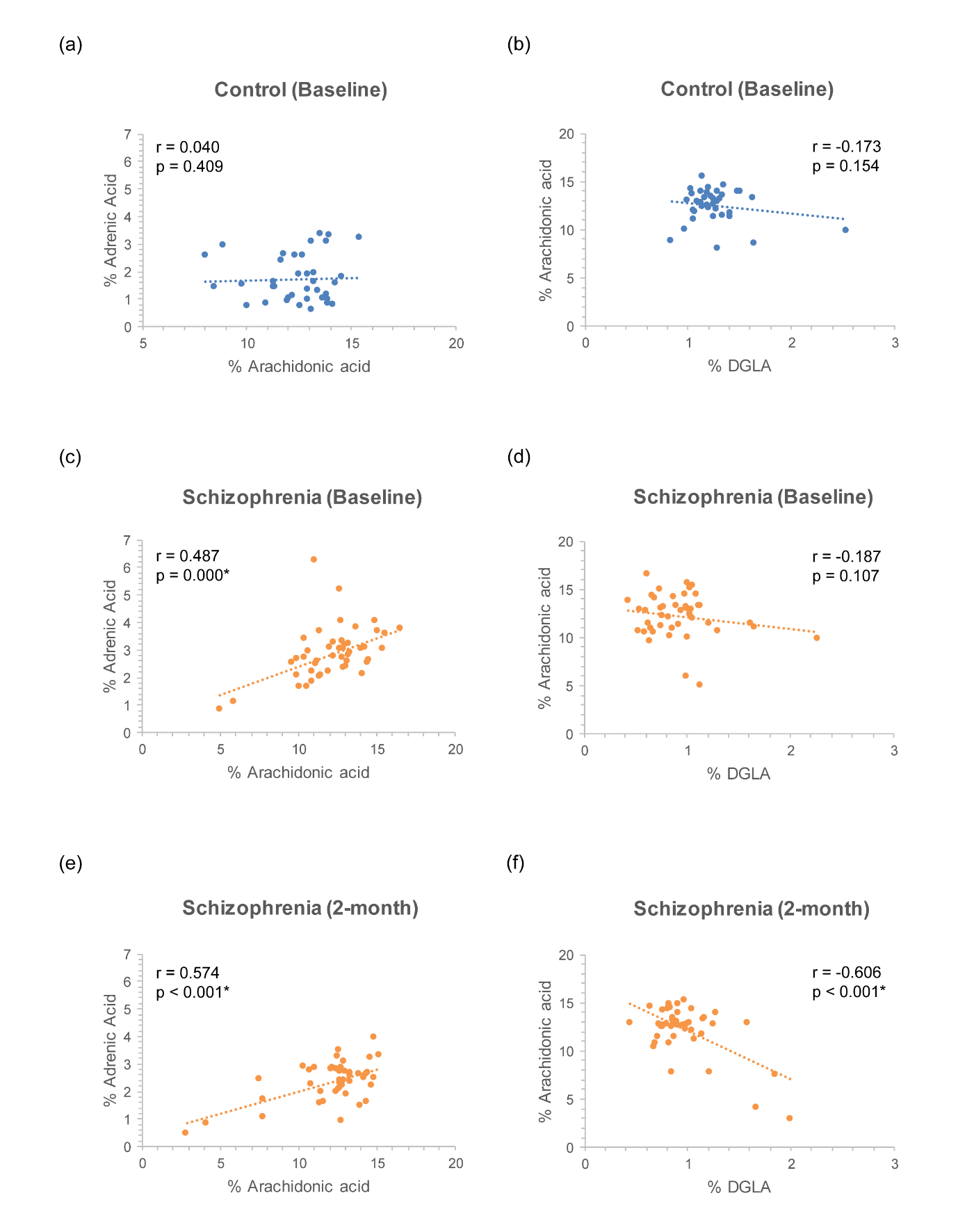


**Fig. S1**. Pearson correlations between the level of arachidonic acid and that of its precursor (dihomo-gamma-linolenic acid, DGLA) and product (adrenic acid) for healthy controls (n = 37) at the baseline (a, b) as well as schizophrenia patients (n = 46) at the baseline (c, d) and at the 2-month follow-up (e and f).

**Table S1.** Niacin scores at a series of time-concentration points in controls and schizophrenia patients at two time

points.

|  | Baseline | | | | |  |  | 2-month follow up | | | | |  |
| --- | --- | --- | --- | --- | --- | --- | --- | --- | --- | --- | --- | --- | --- |
|  | Control  (n = 37) | |  | Schizophrenia  (n = 46) | |  |  | Control  (n = 37) | |  | Schizophrenia  (n = 46) | |  |
| Scores | Mean | (SD) |  | Mean | (SD) | P |  | Mean | (SD) |  | Mean | (SD) | P |
| 5 min |  |  |  |  |  |  |  |  |  |  |  |  |  |
| 0.1 M | 1.95 | (0.57) |  | 1.37 | (0.77) | 0.000 |  | 2.11 | (0.39) |  | 1.43 | (0.66) | 0.000 |
| 0.01 M | 1.38 | (0.72) |  | 0.74 | (0.80) | 0.000 |  | 1.68 | (0.67) |  | 0.76 | (0.67) | 0.000 |
| 0.001M | 0.49 | (0.65) |  | 0.30 | (0.59) | 0.186 |  | 0.86 | (0.71) |  | 0.28 | (0.46) | 0.000 |
| Sum | 3.81 | (1.61) |  | 2.41 | (1.92) | 0.000 |  | 4.65 | (1.49) |  | 2.48 | (1.50) | 0.000 |
| 10 min |  |  |  |  |  |  |  |  |  |  |  |  |  |
| 0.1 M | 2.78 | (0.48) |  | 2.37 | (0.74) | 0.006 |  | 2.97 | (0.16) |  | 2.43 | (0.69) | 0.000 |
| 0.01 M | 2.37 | (0.64) |  | 1.59 | (1.00) | 0.000 |  | 2.68 | (0.53) |  | 1.65 | (0.90) | 0.000 |
| 0.001M | 1.46 | (0.77) |  | 0.91 | (0.94) | 0.006 |  | 1.84 | (0.80) |  | 0.89 | (0.71) | 0.000 |
| Sum | 6.62 | (1.55) |  | 4.87 | (2.47) | 0.000 |  | 7.49 | (1.30) |  | 4.98 | (2.04) | 0.000 |
| 15 min |  |  |  |  |  |  |  |  |  |  |  |  |  |
| 0.1 M | 3.00 | (0.00) |  | 2.83 | (0.44) | 0.010 |  | 3.00 | (0.00) |  | 2.83 | (0.53) | 0.031 |
| 0.01 M | 2.92 | (0.28) |  | 2.33 | (0.97) | 0.000 |  | 2.95 | (0.33) |  | 2.46 | (0.84) | 0.001 |
| 0.001M | 2.43 | (0.65) |  | 1.57 | (1.17) | 0.000 |  | 2.62 | (0.68) |  | 1.61 | (0.93) | 0.000 |
| Sum | 8.35 | (0.82) |  | 6.72 | (2.30) | 0.000 |  | 8.57 | (0.93) |  | 6.89 | (1.99) | 0.000 |
| Composite^a^ | 10.08 | (3.29) |  | 6.48 | (4.67) | 0.000 |  | 11.78 | (3.22) |  | 6.63 | (3.63) | 0.000 |

^a^ Including the scores for the following time-concentration points: 5 min of (0.1M, 0.01M, 0.001M) + 10 min of (0.01M, 0.001M) + 15 min of (0.001M).

**Table S2**. Niacin scores at a series of time-concentration points at the baseline in controls and schizophrenia patients.

|  | Control  (n = 37) | |  | Schizophrenia  (n = 46) | | Control/SZ |
| --- | --- | --- | --- | --- | --- | --- |
| Flush scores | Mean | (SD) |  | Mean | (SD) | Ratio |
| 5 min |  |  |  |  |  |  |
| 0.1 M | 1.95 | (0.57) |  | 1.37 | (0.77)* | 1.42 |
| 0.01 M | 1.38 | (0.72) |  | 0.74 | (0.80)* | 1.86 |
| 0.001M | 0.49 | (0.65) |  | 0.30 | (0.59) | 1.63 |
| 10 min |  |  |  |  |  |  |
| 0.1 M | 2.78 | (0.48) |  | 2.37 | (0.74)* | 1.17 |
| 0.01 M | 2.37 | (0.64) |  | 1.59 | (1.00)* | 1.49 |
| 0.001M | 1.46 | (0.77) |  | 0.91 | (0.94)* | 1.60 |
| 15 min |  |  |  |  |  |  |
| 0.1 M | 3.00 | (0.00) |  | 2.83 | (0.44)* | 1.06 |
| 0.01 M | 2.92 | (0.28) |  | 2.33 | (0.97)* | 1.25 |
| 0.001M | 2.43 | (0.65) |  | 1.57 | (1.17)* | 1.55 |
| Composite score^a^ | 10.57 | (3.51) |  | 6.67 | (5.16)* | 1.58 |

^a^ Including the scores for the following time-concentration points: 5 min of (0.1M, 0.01M, 0.001M) + 10 min of (0.01M, 0.001M) + 15 min of (0.001M).

^*^P < 0.05 for comparison with controls.

**Table S3**. Fatty acid composition (%) of red blood cell at the baseline and the 2-month follow-up of control group.

|  | Baseline  (n = 37) | |  | 2-month follow up (n = 37) | |
| --- | --- | --- | --- | --- | --- |
|  | Mean | (SD) |  | Mean | (SD) |
| C14:0 (Myristic acid) | 0.30 | (0.16) |  | 0.28 | (0.09) |
| C16:0 (Palmitic acid) | 23.49 | (1.42) |  | 24.01 | (1.22) |
| C18:0 (Stearic acid) | 13.34 | (1.12) |  | 13.40 | (1.32) |
| C22:0 (Behenic acid) | 1.29 | (0.22) |  | 1.27 | (0.21) |
| C24:0 (Lignoceric acid) | 0.25 | (0.13) |  | 0.13 | (0.10)**^a^** |
| C14:1 n-5 (Myristoleic acid) | 0.24 | (0.29) |  | 0.17 | (0.29) |
| C16:1 n-7 (Palmitoleic acid) | 0.34 | (0.18) |  | 0.32 | (0.16) |
| C18:1 n-7 (Vaccenic acid) | 0.99 | (0.24) |  | 1.02 | (0.33) |
| C16:1 n-9 (Hypogenic acid) | 0.13 | (0.04) |  | 0.11 | (0.04)**^a^** |
| C18:1 n-9 (Oleic acid) | 14.05 | (1.29) |  | 14.31 | (1.39) |
| C20:1 n-9 (Gondoic acid) | 0.19 | (0.09) |  | 0.22 | (0.09) |
| C24:1 n-9 (Nervoic acid) | 4.85 | (1.60) |  | 3.03 | (0.86)**^a^** |
| C18:2 n-6 (Linoleic acid) | 16.17 | (1.95) |  | 16.48 | (2.10) |
| C18:3 n-6 (γ-Linolenic acid) | 0.28 | (0.08) |  | 0.34 | (0.09)**^a^** |
| C20:2 n-6 (Eicosadienoic acid) | 0.40 | (0.05) |  | 0.40 | (0.10) |
| C20:3 n-6 (Dihomo gamma linolenic acid) | 1.26 | (0.28) |  | 1.00 | (0.27)**^a^** |
| C20:4 n-6 (Arachidonic acid) | 12.43 | (1.71) |  | 12.71 | (1.34) |
| C22:2 n-6 (Docosadienoic acid) | 0.11 | (0.06) |  | 0.15 | (0.08)**^a^** |
| C22:4 n-6 (Adrenic acid) | 1.73 | (0.85) |  | 2.53 | (0.52)**^a^** |
| C22:5 n-6 (Docosapentaenoic acid) | 0.56 | (0.25) |  | 0.51 | (0.15) |
| C18:3 n-3 (α-Linolenic acid) | 0.28 | (0.13) |  | 0.28 | (0.12) |
| C20:5 n-3 (Eicosapentaenoic acid) | 0.62 | (0.27) |  | 0.62 | (0.31) |
| C22:5 n-3 (Docosapentaenoic acid) | 1.70 | (0.66) |  | 1.76 | (0.27) |
| C22:6 n-3 (Docosahexaenoic acid) | 5.01 | (0.96) |  | 4.95 | (0.96) |
| Sum of saturated fatty acids | 38.67 | (2.02) |  | 39.09 | (1.78) |
| Sum of monounsaturated fatty acids | 20.78 | (1.42) |  | 19.18 | (1.55)**^a^** |
| Sum of polyunsaturated fatty acids | 39.78 | (4.26) |  | 41.36 | (3.57) |
| Sum of n-6 fatty acids | 32.94 | (2.49) |  | 34.12 | (2.45)**^a^** |
| Sum of n-3 fatty acids | 7.61 | (1.30) |  | 7.61 | (1.36) |

^a^p < 0.05 (compared the difference between baseline and 2-month follow up by paired t test)

**Table S4**. Fatty acid composition (%) of red blood cell at the baseline and the 2-month follow-up of schizophrenia patients versus the pooled controls.

|  | Schizophrenia (n=46) | | | | | | |  | Pooled control | |
| --- | --- | --- | --- | --- | --- | --- | --- | --- | --- | --- |
|  | Baseline | | |  | 2-month follow up | | |  | (n=37) | |
| Fatty Acids | Mean | (SD) | ES |  | Mean | (SD) | ES |  | Mean | (SD) |
| C14:0 (Myristic acid) | 0.30 | (0.12) | 0.1 |  | 0.34 | (0.15) | 0.4 |  | 0.29 | (0.09) |
| C16:0 (Palmitic acid) | **25.28** | **(2.20)** | **0.8*** |  | **25.50** | **(2.89)** | **0.7*** |  | 23.75 | (1.02) |
| C18:0 (Stearic acid) | 13.13 | (1.72) | -0.2 |  | 13.56 | (2.00) | 0.1 |  | 13.37 | (0.82) |
| C22:0 (Behenic acid) | 1.41 | (0.40) | 0.4 |  | **1.52** | **(0.53)** | **0.6*** |  | 1.28 | (0.20) |
| C24:0 (Lignoceric acid) | **0.11** | **(0.05)** | **-1.1*** |  | **0.12** | **(0.06)** | **-0.9*** |  | 0.19 | (0.08) |
| C14:1 n-5 (Myristoleic acid) | **0.10** | **(0.03)** | **-0.8*** |  | **0.12** | **(0.06)** | **-0.6** |  | 0.21 | (0.20) |
| C16:1 n-7 (Palmitoleic acid) | 0.45 | (0.25) | 0.6 |  | **0.47** | **(0.24)** | **0.7*** |  | 0.33 | (0.13) |
| C18:1 n-7 (Vaccenic acid) | 1.13 | (0.38) | 0.4 |  | 1.09 | (0.44) | 0.2 |  | 1.01 | (0.20) |
| C16:1 n-9 (Hypogenic acid) | **0.10** | **(0.04)** | **-0.5** |  | 0.15 | (0.25) | 0.2 |  | 0.12 | (0.03) |
| C18:1 n-9 (Oleic acid) | 14.45 | (1.64) | 0.2 |  | 14.44 | (1.62) | 0.2 |  | 14.18 | (1.14) |
| C20:1 n-9 (Gondoic acid) | **0.27** | **(0.09)** | **0.9*** |  | **0.25** | **(0.09)** | **0.6*** |  | 0.20 | (0.06) |
| C24:1 n-9 (Nervoic acid) | **2.77** | **(1.02)** | **-1.1*** |  | **3.14** | **(1.05)** | **-0.8*** |  | 3.94 | (0.85) |
| C18:2 n-6 (Linoleic acid) | 15.99 | (2.38) | -0.2 |  | 15.77 | (2.52) | -0.3 |  | 16.33 | (1.64) |
| C18:3 n-6 (γ-Linolenic acid) | **0.42** | **(0.24)** | **0.6*** |  | 0.35 | (0.11) | 0.4 |  | 0.31 | (0.06) |
| C20:2 n-6 (Eicosadienoic acid) | **0.44** | **(0.11)** | **0.4** |  | 0.43 | (0.10) | 0.4 |  | 0.40 | (0.06) |
| C20:3 n-6 (Dihomo gamma linolenic acid) | **0.93** | **(0.33)** | **-0.7*** |  | **0.98** | **(0.31)** | **-0.6*** |  | 1.13 | (0.18) |
| C20:4 n-6 (Arachidonic acid) | 12.22 | (2.22) | -0.2 |  | 12.16 | (2.61) | -0.2 |  | 12.57 | (1.22) |
| C22:2 n-6 (Docosadienoic acid) | **0.15** | **(0.06)** | **0.4** |  | **0.17** | **(0.17)** | **0.3** |  | 0.13 | (0.05) |
| C22:4 n-6 (Adrenic acid) | **2.87** | **(0.94)** | **0.8*** |  | 2.35 | (0.72) | 0.3 |  | 2.13 | (0.57) |
| C22:5 n-6 (Docosapentaenoic acid) | 0.51 | (0.15) | -0.2 |  | 0.52 | (0.23) | -0.1 |  | 0.54 | (0.15) |
| C18:3 n-3 (α-Linolenic acid) | 0.29 | (0.11) | 0.1 |  | 0.28 | (0.08) | 0.0 |  | 0.28 | (0.09) |
| C20:5 n-3 (Eicosapentaenoic acid) | 0.57 | (0.30) | -0.2 |  | 0.52 | (0.22) | -0.4 |  | 0.62 | (0.26) |
| C22:5 n-3 (Docosapentaenoic acid) | 1.75 | (0.42) | 0.1 |  | 1.66 | (0.43) | -0.2 |  | 1.73 | (0.41) |
| C22:6 n-3 (Docosahexaenoic acid) | **4.37** | **(1.23)** | **-0.6*** |  | **4.11** | **(1.23)** | **-0.8*** |  | 4.98 | (0.80) |
| Sum of saturated fatty acids | **40.23** | **(3.20)** | **0.5*** |  | **41.04** | **(3.71)** | **0.7*** |  | 38.88 | (1.31) |
| Sum of monounsaturated fatty acids | **19.27** | **(1.76)** | **-0.5** |  | 19.66 | (2.01) | -0.2 |  | 19.98 | (1.14) |
| Sum of polyunsaturated fatty acids | 40.50 | (4.24) | -0.2 |  | 39.30 | (5.11) | -0.5 |  | *41.14* | *(1.85)* |
| Sum of n-6 fatty acids | 33.52 | (3.20) | 0.0 |  | 32.73 | (4.14) | -0.2 |  | 33.53 | (2.10) |
| Sum of n-3 fatty acids | **6.98** | **(1.75)** | **-0.4** |  | **6.57** | **(1.64)** | **-0.7*** |  | 7.61 | (1.11) |

Pooled control: Mean fatty acids levels of controls at both baseline and 2-month follow up.

ES: Effect size calculated as [(mean difference between schizophrenia and pooled control)/ SD (the whole sample)]

Bold: p < 0.05 for comparison with the pooled controls in linear regressions adjusting for propensity score consisting of age, sex, and smoking.

*Significant after correction for multiple testing by false discovery rate.

**Table S5**. Pearson correlations and Spearman correlations between levels of arachidonic acid (ARA) and its product (adrenic acid) and precursor (dihomo gamma linolenic acid, DGLA), respectively, for controls at the baseline, schizophrenia patients at the baseline, and schizophrenia patients at the 2-month follow up.

|  | *r* (p-value) | | | |
| --- | --- | --- | --- | --- |
|  | Controls |  | Schizophrenia | |
| Types of correlations | Baseline |  | Baseline | 2-month |
| **Pearson correlations between** |  |  |  |  |
| Adrenic acid and ARA | 0.040  (0.409) |  | **0.487**  **(<0.001)** | **0.574**  **(<0.001)** |
| ARA and DGLA | -0.173  (0.154) |  | -0.187  (0.107) | **-0.606**  (**<0.001**) |
| **Spearman correlations between** |  |  |  |  |
| Adrenic acid and ARA | 0.025  (0.885) |  | **0.511**  **(<0.001)** | **0.550**  **(<0.001)** |
| ARA and DGLA | -0.206  (0.236) |  | -0.167  (0.277) | **-0.628**  (**<0.001**) |

Bold: p < 0.05 for the significance testing of the correlation (one-tailed).

**Table S6**. Pearson correlations between n-6 fatty acids (arachidonic acid, its precursor dihomo gamma linolenic acid, and product adrenic acid) and the composite niacin scores for controls at the baseline, schizophrenia patients at the baseline, and schizophrenia patients at the 2-month follow up.

|  | Correlation with the composite niacin scores: *r* (p-value) | | | |
| --- | --- | --- | --- | --- |
|  | Controls |  | Schizophrenia | |
| n-6 fatty acids | Baseline |  | Baseline | 2-month |
| C20:4 n-6 (Arachidonic acid) | **0.317**  **(0.028)** |  | -0.048  (0.376) | 0.239  (0.059) |
| C20:3 n-6 (Dihomo gamma linolenic acid) | **-0.361**  **(0.014)** |  | 0.133  (0.189) | **-0.372**  **(0.006)** |
| C22:4 n-6 (Adrenic acid) | 0.195  (0.124) |  | 0.217  (0.074) | 0.251  (0.051) |

Bold: p < 0.05 for the significance testing of the correlation, which was one-tailed since the primary hypothesis was directional, i.e., higher AA or adrenic acid levels would correlate with more niacin response whereas higher DGLA levels would correlate with less niacin response.

**Table S7**. Spearman correlations between n-6 fatty acids (arachidonic acid, its precursor dihomo gamma linolenic acid, and product adrenic acid) and the composite niacin scores for controls at the baseline, schizophrenia patients at the baseline, and schizophrenia patients at the 2-month follow up.

|  | Correlation with the composite niacin scores: *r* (p-value) | | | |
| --- | --- | --- | --- | --- |
|  | Controls |  | Schizophrenia | |
| n-6 fatty acids | Baseline |  | Baseline | 2-month |
| C20:4 n-6 (Arachidonic acid) | **0.358**  **(0.035)** |  | 0.017  (0.914) | 0.246  (0.116) |
| C20:3 n-6 (Dihomo gamma linolenic acid) | **-0.342**  **(0.044)** |  | 0.114  (0.461) | **-0.427**  **(0.005)** |
| C22:4 n-6 (Adrenic acid) | 0.282  (0.101) |  | 0.203  (0.186) | 0.304  (0.051) |

Bold: p < 0.05 for the significance testing of the correlation, which was one-tailed since the primary hypothesis was directional, i.e., higher AA or adrenic acid levels would correlate with more niacin response whereas higher DGLA levels would correlate with less niacin response.
